# Psychological Inflexibility and Loneliness Mediate the Impact of Stress on Anxiety and Depression Symptoms in Healthcare Students and Early-Career Professionals During COVID-19

**DOI:** 10.3389/fpsyg.2021.729171

**Published:** 2021-09-20

**Authors:** Patricia Bonilla-Sierra, Alexis Manrique-G, Paula Hidalgo-Andrade, Pablo Ruisoto

**Affiliations:** ^1^Faculty of Health Sciences, Universidad Técnica Particular de Loja, Loja, Ecuador; ^2^School of Psychology, Universidad de Las Américas, Quito, Ecuador; ^3^Department of Health Sciences, Public University of Navarra, Pamplona, Spain

**Keywords:** psychological stress, psychological inflexibility, anxiety and depression, loneliness, healthcare professionals

## Abstract

**Background:** The current mental health state of healthcare professionals and students during the COVID-19 pandemic in Ecuador remains understudied and how to improve their mental health is a challenge.

**Objective:** This study aimed to explore the anxiety and depressive symptomatology among healthcare students and professionals in Ecuador and to examine the role of psychological inflexibility, loneliness, and psychological stress as predictors of anxiety and depression symptoms.

**Methods:** A total of 191 undergraduate and graduate healthcare students in clinical practice (early-career healthcare professionals) in Ecuador were surveyed between January and March 2021 using standardized measures of psychological stress (PSS), psychological inflexibility (AAQ), loneliness (UCLA), alcohol consumption (AUDIT-C), and anxiety and depressive symptomatology (PHQ). Macro Process for SPSS (models 4 and 7) were used to test mediation effects.

**Results:** Alcohol consumption varied between men and women and anxiety and depression symptomatology was generally low among the sample. Psychological inflexibility and loneliness mediated the impact of stress on anxiety and depressive mood in participants, regardless of gender and previous personal history of COVID-19.

**Discussion:** Implications of psychological inflexibility and the prevention and coping with stress in healthcare professionals during COVID-19 are further discussed.

## Introduction

Quarantine and lockdown measures in the context of the COVID-19 pandemic have been related to an increased risk of mental health problems (Campion and Knapp, 2018; Campion et al., 2020). Among them, anxiety and depressive symptoms show the highest prevalence, reaching almost one in three people (Salari et al., 2020). Although reports indicate that the psychological impact on the general population is almost double that on health personnel, the anxiety rate of the latter (nurses, doctors) is relevant (Lozano-Vargas, 2020). Moreover, given that physical distancing and self-isolation are the most widespread means to mitigate this pandemic, loneliness is also expected to increase. Social connection helps regulate people's emotions, cope with stress, and remain resilient during adverse times. Conversely, loneliness and social isolation worsen the burden of stress and often produce deleterious effects on mental, cardiovascular, and immune health (Hawkley and Cacioppo, 2010). In fact, perceived loneliness is one of the main predictors of mental health for the general population during the Covid-19 pandemic (González-Sanguino et al., 2021).

However, COVID-19 related distress itself should not be considered a mental disorder. Anguish and anxiety are normal emotions that may allow people to better adapt to the process and can be influenced by different genetic and environmental conditions, as well as previous experiences (Southwick and Charney, 2012; Vinkers et al., 2020). Indeed, from a stress model perspective, the perceptions of uncertainty and uncontrollability are core predictors of increased stress and, therefore, increase risk of anxiety and depression (Vinkers et al., 2020; Batista et al., 2021) or drug abuse or alcohol (Wu et al., 2008; European Monitoring Centre for Drugs Drug Addiction, 2021).

A new paradigm is beginning to emerge in mental health questioning the validity and utility of the medical illness model of mental disorders, shifting from focusing on diseases to using process-based therapies that target the mediators and moderators of those diseases (Hofmann and Hayes, 2019). The most well-known transdiagnostic variable associated with mental health is psychological flexibility (Kashdan and Rottenberg, 2010; Levin et al., 2014; Gloster et al., 2017). Psychological flexibility is defined as the ability to “remain in the present moment and engage in values-based behavior, even in the presence of unpleasant internal experiences” (Kroska et al., 2020, p. 29). People with psychological flexibility feel that existence is meaningful and purposeful, directed and motivated by valued life goals and their importance and show experiential acceptance, accept internal experiences despite being unpleasant, and maintain values-based behaviors (Spatola et al., 2014). Higher psychological flexibility predicts mental health and healthy behaviors, promotes well-being, and could stimulate resilience (Kroska et al., 2020; Hernández-López et al., 2021). On the other hand, psychological inflexibility refers to a rigid tendency to control aversive private events, such as memories, feelings, or thoughts, by avoiding or escaping from them (Kashdan and Rottenberg, 2010; Levin et al., 2014; Gloster et al., 2017). Thus, psychological inflexibility represents a form of generalized psychological vulnerability (Kashdan et al., 2006; Kashdan and Rottenberg, 2010; Levin et al., 2014) associated with greater depressive symptoms (Kato, 2016), anxiety, psychopathological conditions, and an increased risk for the deterioration of mental (Hernández-López et al., 2021) and physical health (Spatola et al., 2014).

Healthcare professionals are considered one of the most vulnerable groups for negative mental effects from COVID-19 (Siddaway, 2020), particularly anxiety and depressive symptoms (Badahdah et al., 2020). This situation could be exacerbated by prolonged quarantine, fear of infection, frustration, boredom, inadequate supplies, false information, the insecurity of an unclear and disease-free future, increased workload, lack of adequate protection, fear of becoming infected and infecting their loved ones, social stigma, loneliness, misinformation, among others (Brooks et al., 2020). Unexperienced professionals, as well as students with direct clinical experience during the pandemic, might also suffer some of these consequences. For example, a study with medical students in China during the COVID-19 outbreak showed that the pandemic negatively impacted their stress and feelings of loneliness (Zheng et al., 2021). The same study revealed that loneliness mediated the relationship between perceived mental stress and influence on career choice.

Like other countries, Ecuador has also been affected by the pandemic and its population has suffered personal and economic losses. Due to COVID-19, Ecuador has a high mortality rate and it is one of the most affected territories in Latin America (Servicio Nacional de Gestión de Riesgos y Emergencias, 2021). The pandemic collapsed its health system, forcing physical distancing and changing the lifestyle of its people. Studies during the pandemic show that healthcare workers in Ecuador presented moderate levels of burnout and compassion fatigue (Cuartero-Castañer et al., 2021). Also, around 20% of people in epidemiological surveillance for COVID-19 showed moderate to severe symptoms of depression and anxiety (Paz et al., 2020). Regarding sociodemographic factors, being female and living in the coastal region were associated with more anxiety and depression symptoms (Paz et al., 2020). Similar results were found in the general population where between 10 and 19% of the people showed severe or extremely severe symptoms of depression, anxiety, and stress (Tusev et al., 2020). However, the prevalence of mental health problems such as anxiety and depressive symptomatology among healthcare students in clinical practice and professionals (early career professionals) during COVID-19 in Ecuador remains unknown, as well as the role of transdiagnostic variables, such as psychological inflexibility or loneliness, that might mediate the negative impact of psychological stress. This study aimed to explore the prevalence of anxiety and depressive symptomatology among early-career healthcare professionals in Ecuador and to examine the role of psychological inflexibility and loneliness on the impact of psychological stress in anxious and depressive symptomatology among this population.

## Methods

### Participants

This article analyses the data from a convenience sample of 191 early-career healthcare professionals which include senior undergraduate and graduate students of healthcare careers (medicine, nursing, and clinical psychology) from two private universities in Ecuador. To be part of the study, participants had to be enrolled as undergraduate or graduate students and have direct supervised clinical experience in their field during the COVID-19 pandemic.

### Measures

In addition to sociodemographic variables such as gender, age, marital status, workload (h/week), professional category, specific training in COVID-19, and personal history of COVID-19 (diagnosis or presence of compatible symptoms), the following standardized scales were assessed in Spanish:

*UCLA Loneliness Scale-Revised, short version* (Hughes et al., 2004). This is a brief three-item scale that evaluates the subjective feeling of loneliness, understood as the perception of less-than-desired availability of social support. We used a translation previously used in Ecuador (Ruisoto et al., 2016; López et al., 2019). Participants respond based on their agreement with statements in a Likert-type response (1 = “hardly ever,” 2 = “sometimes,” and 3 = “often”). Total scores range from 0 to 9. Higher scores indicate a greater feeling of loneliness or a lack of social support. The Cronbach's alpha coefficient for internal consistency was α = 0.857.

*Perceived Stress Scale (PSS-14*). We used the Ecuadorian version (Ruisoto et al., 2020). This scale has 14 items that assess the degree to which people perceive a lack of control in their daily lives. Participants respond to a five-point Likert-type scale ranging from 0 (never) to 4 (very often). Total scores range from 0 to 56. Higher scores indicate higher levels of stress. It has good psychometric properties and correlates with cortisol measurements in the blood and saliva. Cronbach's alpha coefficient for internal consistency reliability was α = 0.883.

*Avoidance and Action Questionnaire (AAQ-7*, Bond et al., 2011). This is the most widely used general measure of psychological inflexibility, defined as rigidity in the handling of emotions or unpleasant internal events. We used a translation previously used in Ecuador (Ruisoto et al., 2020). It consists of seven items and participants respond to a seven-point Likert-type scale, from 1 (never) to 7 (always). Scores range from 7 to 49. Higher scores indicate higher psychological inflexibility. The reliability of the scale was α = 0.944.

*Patient Health Questionnaire of Depression and Anxiety (PHQ-4*, Löwe et al., 2010). This questionnaire assesses depression and anxiety associated with symptom burden, functional impairment, and disability we applied a version previously used in Ecuadorian population (López et al., 2019). The scores range from 0 to 12. A higher score indicates a greater anxiety and depression symptoms. The Cronbach's alpha of the questionnaire was α = 0.884.

*Alcohol Use Disorders Identification Test (AUDIT-C*, Bush et al., 1998; Bradley et al., 2003). It is composed of the three first items of the full 10 items version of the AUDIT. It is used to screen for alcohol consumption. Participants respond based on the frequency or amount of alcohol participants consume. Scores below 3 points are consistent with normal alcohol consumption. We used a version validated in Ecuador (López et al., 2019). The Cronbach's alpha coefficient for internal consistency reliability was α = 0.775.

### Design and Procedure

A descriptive quantitative cross-sectional study was conducted. Data were collected between January and March 2021 through a recruitment email distributed to the target audience by the universities' mail servers. The email contained a link to an anonymous online survey on SurveyMonkey. At the time of the study, classes were still remote and there were several mobility restrictions due to the COVID-19 pandemic. The study was approved by the Human Research Ethics Committee (*Comité de Ética de Investigación en Seres Humanos, CEISH*) of the Ministry of Public Health of the Republic of Ecuador (No. 014-2020) and was conducted according to the principles expressed in the Declaration of Helsinki (World Medical Association, 2013). Informed consent was displayed on the first page of the survey. After reading it, those who voluntarily wanted to participate had to accept it before continuing to the rest of the survey which took around 15 min to complete.

### Data Analysis

All data analyses were performed using the Statistical Package for the Social Sciences (SPSS) version 21 for Mac (IBM, Madrid, Spain). The descriptive analysis of the sample included the means and standard deviations (M ± SD) for the quantitative variables, while frequencies and percentages were used for the nominal variables. Student's *t*-test was used to analyze differences between men and women in the measured variables. The effect size was calculated using Cohen's *d*. Independent hierarchical multiple regression models were also applied to examine the effects of sociodemographic (Step 1) and psychological variables (Step 2) on anxiety and depressive symptoms among healthcare professionals. A standard method of entry was used for variable selection (enter method); thus, the effect of all independent variables was analyzed at the same time. The detection of multicollinearity was performed using the Variance Inflation Factor (VIF), with VIF > 5 as the cut-off point for the diagnosis of collinearity (Sheather, 2009). For multiple regressions, the R2 was obtained. Additionally, residual plots were used to assess the goodness of fit for the regression model. Finally, the indirect effect of both psychological inflexibility on the effect of psychological stress on anxiety and depressive mood were examined using the bootstrap method with the Process macro version 3.3 (Hayes, 2018) for SPSS (model 4). The number of bootstrap samples was set to 10,000. A complementary mediational triangle was used to visually display the mediation effects (Baron and Kenny, 1986). The significance level was set to *p* < 0.05.

## Results

### Sample Description

A total of 191 early-career healthcare professionals in Ecuador participated in the study. These included senior undergraduate and regular graduate students in healthcare careers such as medicine, nursing, and clinical psychology, with direct supervised experience caring for patients either in person or by telemedicine or telepsychology during the COVID-19 pandemic. From the total sample, 29.8% were men and 70.2% women. The average response rate was 47.8%. Age ranged from 18 to 47 years old, with an average age of 26.29 years (*SD* = 5.48). Age for men was M = 26.5 years (*SD* = 6.02) and, for women, *M* = 26.34 years (*SD* = 5.5.1). A total of 70.2% of the sample was single, 25.1% married or common law couple, and 4.7% separated or divorced.

A total of 29.8% of the sample worked full-time and 71.2% part time (34.6% employed with <10 h per week). Most participants worked in the public sector (72.7%), with 27.3% working in private institutions. A total of 39.3% failed to report any specific training about COVID-19 (60.7% did).

Table 1 shows the descriptive statistics and correlations of the variables. Age was negatively related to psychological inflexibility, anxiety and depressive symptoms, as well as to alcohol consumption. The outcome variables (perceived stress, loneliness, psychological inflexibility, and anxiety and depression symptoms) showed a significant positive correlation, while alcohol consumption was only related to perceived stress.

**Table 1:** Descriptive statistics and correlations for main study variables.

**Variable**	***M***	***SD***	**1**	**2**	**3**	**4**	**5**	**6**
1. Psychological stress	27.52	8.79	–					
2. Loneliness	7.61	2.43	0.48^**^	–				
3. Psychological inflexibility	23.22	10.18	0.68^**^	0.49^**^	–			
4. Anxiety and depression symptoms	4.62	3.39	0.72^**^	0.46^**^	0.71^**^	–		
5. Alcohol consumption	5.96	2.54	0.15^*^	0.08	0.11	0.10	–	
6. Age	26.29	5.48	−0.10	−0.10	−0.22^**^	−0.14^*^	-0.18^**^	–

* *p < 0.05*.

** *p < 0.01*.

Gender differences in the outcome variables for this study are shown in Table 2. Only the difference in alcohol consumption varied significantly, and men showed higher consumption than women. Regarding the history of COVID-19, only 9.52% of the sample have tested positive for the virus. However, this value may underestimate the real number due to asymptomatic patients and a lack of general screening tests. Table 3 shows the differences between early-career healthcare professionals with and without a positive history of COVID-19 diagnosis. There were no statistically significant differences.

**Table 2:** Gender differences in outcome variables.

**Variables**	**Males**	**Females**	**t**	***p***	***d***
	**M (SD)**	**M (SD)**			
	**(*n* = 57)**	**(*n* = 134)**			
Psychological stress	26.01 (9.57)	28.05 (8.37)	−0.146	0.146	0.227
Loneliness	7.08 (2.78)	7.79 (2.26)	−1.851	0.092	0.280
Psychological inflexibility	21.19 (10.08)	24.01 (10.13)	−1.752	0.083	0.279
Anxiety and depressive symptomatology	4.66 (3.3)	4.61 (3.4)	0.090	0.929	0.015
Alcohol consumption	6.61 (2.7)	5.66 (2.45)	2.375	0.019^*^	0.368

* *Cohen's d*.

**Table 3:** Differences in outcome variables by history of COVID-19.

**Variables**	**History of COVID-19**	**No history of COVID-19**	**t**	***p***	d^*^
	**M (SD) (*n* = 18)**	**M (SD) (*n* = 171)**			
Psychological stress	27.78 (7.18)	27.41 (8.93)	0.166	0.868	0.045
Loneliness	7.16 (1.94)	7.63 (2.48)	−0.765	0.445	0.211
Psychological inflexibility	24 (9.91)	23.09 (10.22)	0.359	0.360	0.092
Anxiety and depressive symptomatology	5.83 (3.74)	4.5 (3.31)	1.595	0.112	0.381
Alcohol consumption	6.61 (1.94)	5.66 (2.61)	−0.779	0.437	0.413

* *Effect-size r for Cohen's d*.

### Hierarchical Regression Analysis

Hierarchical multiple regression (Table 4) showed that the sociodemographic variables age and history of COVID-19 failed to predict anxiety and depressive symptoms (step 1). However, when adding the psychological variables, gender was significant, as well as the transdiagnostic psychological variables. Stress, psychological inflexibility, and loneliness predicted anxiety and depressive mood (step 2). Alcohol consumption was not a significant variable in the analysis.

**Table 4:** Hierarchical regression analysis for anxiety and depressive symptomatology.

**Regression models (steps and predictors)**	**b**	**Standard error**	** $\eta_{\text{p}}^{2}$ **	**Confidence interval (95%)**	***p***	**VIF**
Step 1 (*R^2^* =0.043)						
Gender (men/women)	0.124	0.547	0.000	−0.956/−1.204	0.821	1.032
Age (years)	−0.081	0.046	0.016	−0.171/0.009	0.078	1.032
History of COVID-19	−1.439	0.836	0.016	−3.089/0.211	0.087	1.010
Alcohol consumption	0.120	0.100	0.007	−0.077 /0.316	0.232	1.063
Step 2 (*R^2^* =0.624)						
Gender (men/women)	−0.810	0.346	0.029	−1.493/−0.127	0.020	1.062
Age (years)	0.005	0.029	0.000	−0.063 /0.052	0.086	1.084
History of COVID-19	−1.146	0.523	0.009	−2.178 /−0.113	0.300	1.018
Alcohol consumption	−0.030	0.063	0.000	−0.155/0.094	0.630	1.090
Psychological stress	0.163	0.025	0.088	0.114 /0.212	<0.001	2.004
Loneliness	0.124	0.075	0.006	−0.023/0.272	0.023	1.402
Psychological inflexibility	0.131	0.022	0.074	0.088/0.173	<0.001	2.067

“*b” = unstandardized coefficient*.

### Mediation Analysis

**Figure 1:**
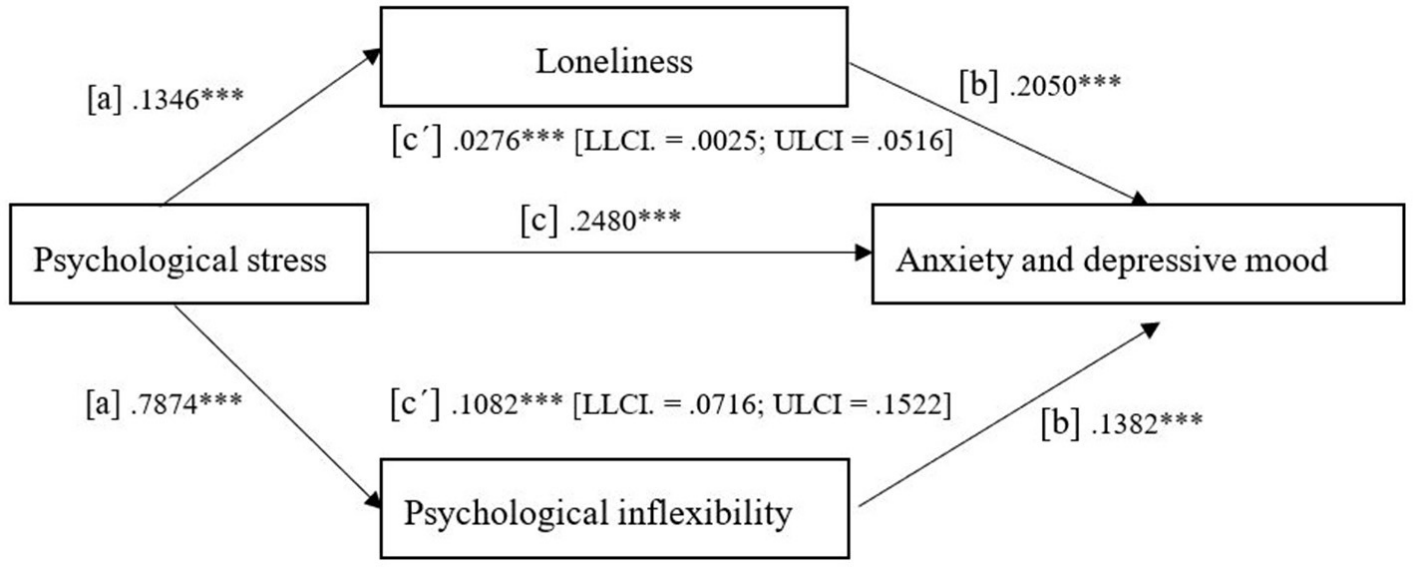
The unstandardized regression coefficients for the mediating effect of psychological inflexibility and loneliness on the relationship between psychological stress and anxious and depressive mood. ***p < 0.001.

Psychological inflexibility and loneliness mediated the impact of stress on anxiety and depressive symptoms in early-career healthcare professionals during the COVID-19 pandemic (Figure 1). This finding was consistent regardless of gender and positive or negative diagnosis of COVID-19.

## Discussion

The COVID-19 pandemic has strained health systems and their workers. Although a previous study in Ecuador indicates that healthcare professionals show average levels of burnout and compassion fatigue (Cuartero-Castañer et al., 2021), we must advocate for better efforts to prevent other negative consequences of the pandemic. In Spain, for example, suicidal thoughts and behaviors have been common among healthcare professionals due to different factors such as lack of communication, coordination, personnel, and supervision (Mortier et al., 2021). Despite growing concern about mental health, most healthcare professionals, even those working in COVID-19 units, fail to receive any training in providing mental healthcare. Indeed, healthcare students and professionals will benefit, not only from raising awareness about the mental health impact of COVID-19 but from learning how to intervene to reduce stress (Folkman and Greer, 2000; Duan and Zhu, 2020).

The current study explored the anxiety and depressive symptomatology among early-career healthcare professionals in Ecuador. Results indicate that, on average there are low levels of these symptoms. We also examined how stress, psychological inflexibility, and loneliness relate to anxiety and depressive symptomatology in early-career healthcare professionals. One main result of this study is the double mediation model which shows the key role of psychological inflexibility and loneliness as mediators of the negative impact of psychological stress on anxiety and depressive symptomatology among the sample. Interestingly, gender and a COVID-19 diagnosis did not influence this mediation. In other cultural contexts such as the United States (Kroska et al., 2020), Italy (Pakenham et al., 2020), and Sweden (McCracken et al., 2021), psychological flexibility has also been identified as a resilience factor and inflexibility as a predictor of peritraumatic stress. Our results highlight the cross-cultural importance of psychological flexibility and the role of transdiagnostic processes in mental health, consistent with pre-pandemic studies (Kashdan and Rottenberg, 2010; Levin et al., 2014; Gloster et al., 2017; Hayes et al., 2019; Hofmann and Hayes, 2019).

Our results contribute to the literature showing that loneliness is a significant predictor of anxiety and depressive symptomatology in the studied context. These agree with previous studies that indicate that loneliness is a risk factor for illnesses such as dementia (Livingston et al., 2020) and that it is related to decreased physical and health, increased mortality, as well as decreased cognitive functioning (Hawkley and Cacioppo, 2010). It has also been found that dispositional loneliness predicts depressive and anxiety symptomatology and, along with fear of COVID-19, it is a risk factor for these symptoms (Rossi et al., 2020).

Furthermore, our model showed that loneliness, along with psychological inflexibility, mediated the negative impact of psychological stress on anxiety and depressive symptomatology among early-career healthcare professionals. This result is of great interest, especially considering that distancing and lockdown served a bigger purpose -to stop the spread of the virus- and thus, the possible perception of loneliness may have been part of the required adaptation to the circumstances (Walsh, 2020) and may have adopted a different meaning. Additionally, all guidelines suggested avoiding isolation and keeping in touch with loved ones through technology (e.g., World Health Organization, 2020; CDC, 2021). Also, given the sample composition, it is fair to assume that participants had access to interact with others through classes, professional practicum, clinical supervision, and work. Despite these multiple opportunities, it seems that the loneliness experienced during this time still had detrimental effects. Future research and interventions for this population must promote interaction and social support, proven-effective strategies to reduce loneliness and thus reduce stress and promote well-being (Elmer et al., 2020).

Moreover, our results emphasize the importance of psychosocial factors to promote well-being and prevent mental health problems in early-career healthcare professionals in Ecuador. They point to integrating approaches to comprehend, avoid, and treat diseases in complex circumstances. Our results are consistent with other authors who go beyond COVID-19 as a pandemic and illustrate the need to frame it as a syndemic, an approach that reveals other interactions between conditions, states, and individuals for prognosis, treatment, and policy (Horton, 2020). This way, health policy could include the existing inequalities and reveal the importance of the interactions between biological and social factors to effectively prevent and respond to other illnesses while facing COVID-19 and its consequences (Horton, 2020).

Collaboration and mutual aid should become widespread in response to COVID-19 urging us to act for the common good (Carter et al., 2015; Bavel et al., 2020). Other countries such as Ecuador could review international efforts and implement them for their own workers. In Spain, for example, some hospitals developed psychological intervention programs for healthcare professionals treating COVID-19 patients (Priede et al., 2021). These included individual and group psychoeducation, mindfulness, and cognitive-behavioral techniques to improve emotional regulation, reduce physiological arousal, and to improve communication skills. Although authors suggest reviewing the efficacy of the programs, their benefits are highlighted. Our contribution to these psychological interventions is to include improving psychological flexibility as an explicit objective.

Special attention should be placed on more vulnerable populations. Like other studies in the same cultural context (Paz et al., 2020; Cuartero-Castañer et al., 2021; Mautong et al., 2021), our results show that younger people are more at risk. We found that younger age correlated to higher psychological inflexibility, more anxiety and depression symptoms, and more alcohol consumption. Consistent with the same research, females report higher levels of stress, although the level of significance was *p* = 0.05. This study also indicates that being female is a significant predictor of anxiety and depressive symptoms. This sociodemographic risk factor may be explained by all the restriction measures in place (e.g., remote education, curfews) that may have added to the already existing gender inequalities in the country (Castellanos-Torres et al., 2020). On the other hand, male respondents in the sample reported higher alcohol consumption than females. Even though research from other cultural contexts shows that people in educational, welfare, and health fields had less likelihood for increased drinking compared to other sectors during the pandemic (Oksanen et al., 2021), this information should also be taken into consideration when planning strategies to help early-career healthcare workers face the pandemic and its consequences.

Despite the relevance of this study, its limitations must be acknowledged. First, the results should be considered with caution since their conclusions are based on self-reported measures on an online survey. Future research should explore whether these results can be replicated in other populations with different sampling methods. Second, the participants were undergraduate and graduate students in clinical practice during the pandemic from two private universities in two different cities in the country; we did not systematically collect data from all early-career healthcare professionals in Ecuador, thus the small sample size and composition. Third, given the nature of the study, all participants had supervision from their universities and/or from the sites they attended patients. This may have affected their responses and a future comparative study between early-career professionals with and without supervision is needed to understand the role of this variable. Finally, the cross-sectional design of the study implies some limitations that only future longitudinal studies could overcome. Nevertheless, by predicting anxiety and depressive mood and analyzing the mediating effect of psychological inflexibility on the relationship between them and stress in an Ecuadorian sample, this study makes a novel contribution. In sum, mental health should be part of an integrated response to COVID-19 with long-lasting positive effects that may outlast the pandemic. To reach this goal, it is a priority to develop psychological interventions to meet the mental health problems in both COVID-patients and healthcare professionals (Duan and Zhu, 2020). Those measures should include improving psychological flexibility which is negatively related to burnout and anxiety and positively related to life satisfaction in healthcare workers (Montaner et al., 2021). Such interventions can mitigate the detrimental effects that stress and stressful situations as the pandemic can have on mental health and the professional quality of life of early-career healthcare workers.

## Data Availability Statement

The raw data supporting the conclusions of this article will be made available by the authors, without undue reservation.

## Ethics Statement

The studies involving human participants were reviewed and approved by Human Research Ethics Committee (Comité de Ética de Investigación en Seres Humanos, CEISH) of the Ministry of Public Health of the Republic of Ecuador (No. 014-2020). The patients/participants provided their written informed consent to participate in this study.

## Author Contributions

PB-S has contributed to the funding and design of the study. PB-S, AM-G, and PH-A have contributed to data collection and drafting the manuscript. PR has contributed to the selection of measures, data curation, statistical analysis and writing of the manuscript. PH-A has contributed to writing, reviewing, and editing the manuscript. Finally, all authors have read and agreed to the published version of the manuscript.

## Funding

This study has been funded by the Universidad Técnica Particular de Loja, under the project PROY-INV-CCSAL_2020_2731 leaded by PB-S.

## Conflict of Interest

The authors declare that the research was conducted in the absence of any commercial or financial relationships that could be construed as a potential conflict of interest.

## Publisher's Note

All claims expressed in this article are solely those of the authors and do not necessarily represent those of their affiliated organizations, or those of the publisher, the editors and the reviewers. Any product that may be evaluated in this article, or claim that may be made by its manufacturer, is not guaranteed or endorsed by the publisher.
