# Supplementary material for: Psychological Inflexibility and Loneliness Mediate the Impact of Stress on Anxiety and Depression Symptoms in Healthcare Students and Early-Career Professionals During COVID-19
Source: Front Psychol. 2021 Sep 20;12:729171. doi: 10.3389/fpsyg.2021.729171 (PMC8491304; doi:10.3389/fpsyg.2021.729171)

IBM SPSS Web Report - residual plot.spv

Log

Log - Log - August 4, 2021

```
REGRESSION
  /DESCRIPTIVES MEAN STDDEV CORR SIG N
  /MISSING LISTWISE
  /STATISTICS COEFF OUTS CI(95) R ANOVA COLLIN TOL CHANGE
  /CRITERIA=PIN(.05) POUT(.10)
  /NOORIGIN
  /DEPENDENT PHQT
  /METHOD=ENTER Sexo Edad COVID AUDITC
  /METHOD=ENTER PSST UCLAT AAQT
  /SCATTERPLOT=(*ZRESID ,*ZPRED)
  /RESIDUALS HISTOGRAM(ZRESID) NORMPROB(ZRESID).
```

Regression

Regression - Descriptive Statistics - August 4, 2021

Descriptive Statistics

	Mean	Std. Deviation	N
PHQT	4.6203	3.39358	187
Sexo	1.7059	.45687	187
Edad	26.29	5.481	187
COVID	1.9037	.29573	187
AUDITC	5.9572	2.54387	187
PSS10	27.5187	8.78742	187
UCLA	7.6096	2.43463	187
AAQT	23.2246	10.17708	187

Regression

Regression - Correlations - August 4, 2021

Correlations

		PHQT	Sexo	Edad	COVID	AUDITC	PSS10	UCLA	AAQT
Pearson Correlation	PHQT	1.000	-.010	-.144	-.117	.103	.718	.462	.711
	Sexo	-.010	1.000	.030	.068	-.159	.108	.128	.116
	Edad	-.144	.030	1.000	-.016	-.177	-.104	-.101	-.217
	COVID	-.117	.068	-.016	1.000	.059	-.010	.060	-.025
	AUDITC	.103	-.159	-.177	.059	1.000	.149	.080	.109
	PSS10	.718	.108	-.104	-.010	.149	1.000	.481	.680
	UCLA	.462	.128	-.101	.060	.080	.481	1.000	.485
	AAQT	.711	.116	-.217	-.025	.109	.680	.485	1.000
Sig. (1-tailed)	PHQT	.	.446	.025	.055	.081	.000	.000	.000
	Sexo	.446	.	.340	.178	.015	.071	.040	.057
	Edad	.025	.340	.	.416	.008	.078	.085	.001
	COVID	.055	.178	.416	.	.212	.448	.209	.367
	AUDITC	.081	.015	.008	.212	.	.021	.139	.069
	PSS10	.000	.071	.078	.448	.021	.	.000	.000
	UCLA	.000	.040	.085	.209	.139	.000	.	.000
	AAQT	.000	.057	.001	.367	.069	.000	.000	.
N	PHQT	187	187	187	187	187	187	187	187
	Sexo	187	187	187	187	187	187	187	187
	Edad	187	187	187	187	187	187	187	187
	COVID	187	187	187	187	187	187	187	187
	AUDITC	187	187	187	187	187	187	187	187
	PSS10	187	187	187	187	187	187	187	187

IBM SPSS Web Report - residual plot.spv

Regression

Regression - Variables Entered/Removed - August 4, 2021

Variables Entered/Removed[a]

Model	Variables Entered	Variables Removed	Method
1	AUDITC, COVID, Sexo, Edad[b]	.	Enter
2	UCLA, PSS10, AAQT[b]	.	Enter

a. Dependent Variable: PHQT

b. All requested variables entered.

Regression

Regression - Model Summary - August 4, 2021

Model Summary[c]

Model	R	R Square	Adjusted R Square	Std. Error of the Estimate	Change Statistics				
					R Square Change	F Change	df1	df2	Sig. F Change
1	.206[a]	.043	.021	3.35694	.043	2.021	4	182	.093
2	.796[b]	.634	.620	2.09240	.592	96.485	3	179	.000

a. Predictors: (Constant), AUDITC, COVID, Sexo, Edad

b. Predictors: (Constant), AUDITC, COVID, Sexo, Edad, UCLA, PSS10, AAQT

c. Dependent Variable: PHQT

Regression

Regression - ANOVA - August 4, 2021

ANOVA[a]

Model		Sum of Squares	df	Mean Square	F	Sig.
1	Regression	91.080	4	22.770	2.021	.093[b]
	Residual	2050.963	182	11.269		
	Total	2142.043	186			
2	Regression	1358.354	7	194.051	44.322	.000[c]
	Residual	783.689	179	4.378		
	Total	2142.043	186			

a. Dependent Variable: PHQT

b. Predictors: (Constant), AUDITC, COVID, Sexo, Edad

c. Predictors: (Constant), AUDITC, COVID, Sexo, Edad, UCLA, PSS10, AAQT

IBM SPSS Web Report - residual plot.spv

Regression

Regression - Coefficients - August 4, 2021

Coefficients[a]

Model		Unstandardized Coefficients		Standardized Coefficients	t	Sig.	95.0% Confidence Interval for B		Collinearity Statistics	
		B	Std. Error	Beta			Lower Bound	Upper Bound	Tolerance	VIF
1	(Constant)	8.562	2.310		3.706	.000	4.003	13.120		
	Sexo	.124	.547	.017	.227	.821	-.956	1.204	.969	1.032
	Edad	-.081	.046	-.131	-1.772	.078	-.171	.009	.969	1.032
	COVID	-1.439	.836	-.125	-1.721	.087	-3.089	.211	.990	1.010
	AUDITC	.120	.100	.090	1.199	.232	-.077	.316	.941	1.063
2	(Constant)	.032	1.530		.021	.984	-2.988	3.051		
	Sexo	-.810	.346	-.109	-2.339	.020	-1.493	-.127	.941	1.062
	Edad	-.005	.029	-.008	-.173	.863	-.063	.052	.922	1.084
	COVID	-1.146	.523	-.100	-2.189	.030	-2.178	-.113	.983	1.018
	AUDITC	-.030	.063	-.023	-.483	.630	-.155	.094	.918	1.090
	PSS10	.163	.025	.422	6.595	.000	.114	.212	.499	2.004
	UCLA	.124	.075	.089	1.668	.097	-.023	.272	.713	1.402
	AAQT	.131	.022	.392	6.024	.000	.088	.173	.484	2.067

a. Dependent Variable: PHQT

Regression

Regression - Excluded Variables - August 4, 2021

Excluded Variables[a]

Model		Beta In	t	Sig.	Partial Correlation	Collinearity Statistics		
						Tolerance	VIF	Minimum Tolerance
1	PSS10	.722[b]	13.965	.000	.720	.953	1.049	.918
	UCLA	.463[b]	7.065	.000	.465	.964	1.038	.934
	AAQT	.720[b]	13.510	.000	.709	.927	1.078	.927

a. Dependent Variable: PHQT

b. Predictors in the Model: (Constant), AUDITC, COVID, Sexo, Edad

Regression

Regression - Collinearity Diagnostics - August 4, 2021

Collinearity Diagnostics[a]

Model	Dimension	Eigenvalue	Condition Index	Variance Proportions							
				(Constant)	Sexo	Edad	COVID	AUDITC	PSS10	UCLA	AAQT
1	1	4.761	1.000	.00	.00	.00	.00	.01			
	2	.148	5.676	.00	.06	.02	.00	.75			
	3	.054	9.422	.00	.72	.26	.01	.03			
	4	.029	12.736	.01	.13	.43	.45	.12			
	5	.008	23.832	.98	.10	.28	.54	.09			
2	1	7.497	1.000	.00	.00	.00	.00	.00	.00	.00	.00
	2	.174	6.557	.00	.01	.03	.01	.07	.03	.02	.21
	3	.148	7.106	.00	.06	.02	.00	.72	.00	.00	.00
	4	.057	11.458	.00	.20	.00	.00	.01	.00	.79	.16
	5	.052	12.007	.00	.57	.21	.00	.05	.06	.14	.02
	6	.035	14.588	.00	.04	.00	.05	.00	.83	.01	.53
	7	.028	16.336	.01	.06	.42	.44	.10	.07	.03	.05

IBM SPSS Web Report - residual plot.spv

Regression

Regression - Residuals Statistics - August 4, 2021

Residuals Statistics[a]

	Minimum	Maximum	Mean	Std. Deviation	N
Predicted Value	-2.0899	11.7093	4.6203	2.70240	187
Residual	-5.34098	7.18556	.00000	2.05265	187
Std. Predicted Value	-2.483	2.623	.000	1.000	187
Std. Residual	-2.553	3.434	.000	.981	187

a. Dependent Variable: PHQT

Charts

Charts - *zresid Histogram - August 4, 2021

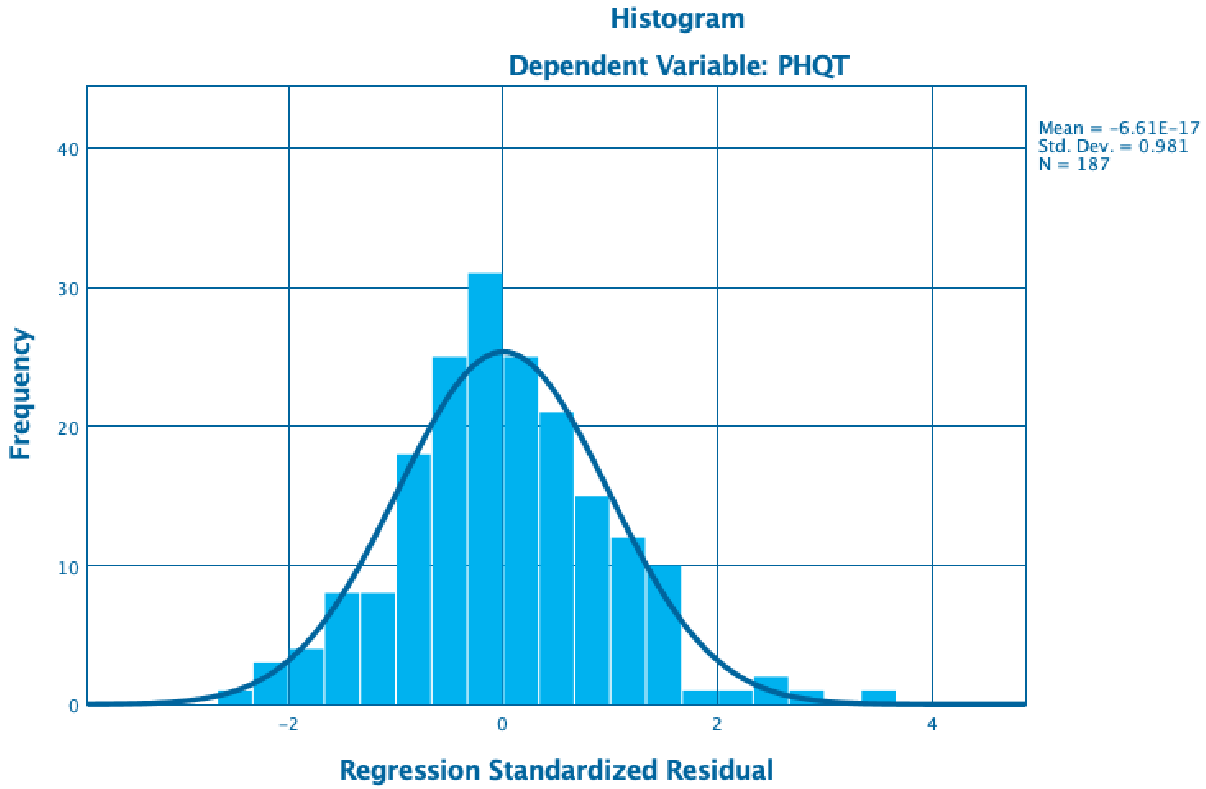

IBM SPSS Web Report - residual plot.spv

Charts

Charts - *zresid Normal P-P Plot - August 4, 2021

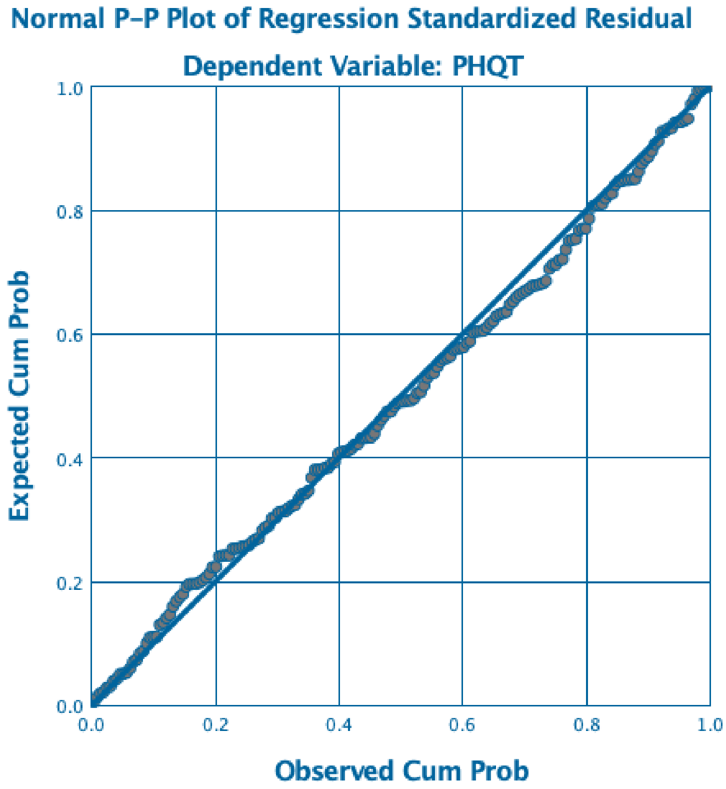

Charts

Charts - *zresid by *zpred Scatterplot - August 4, 2021

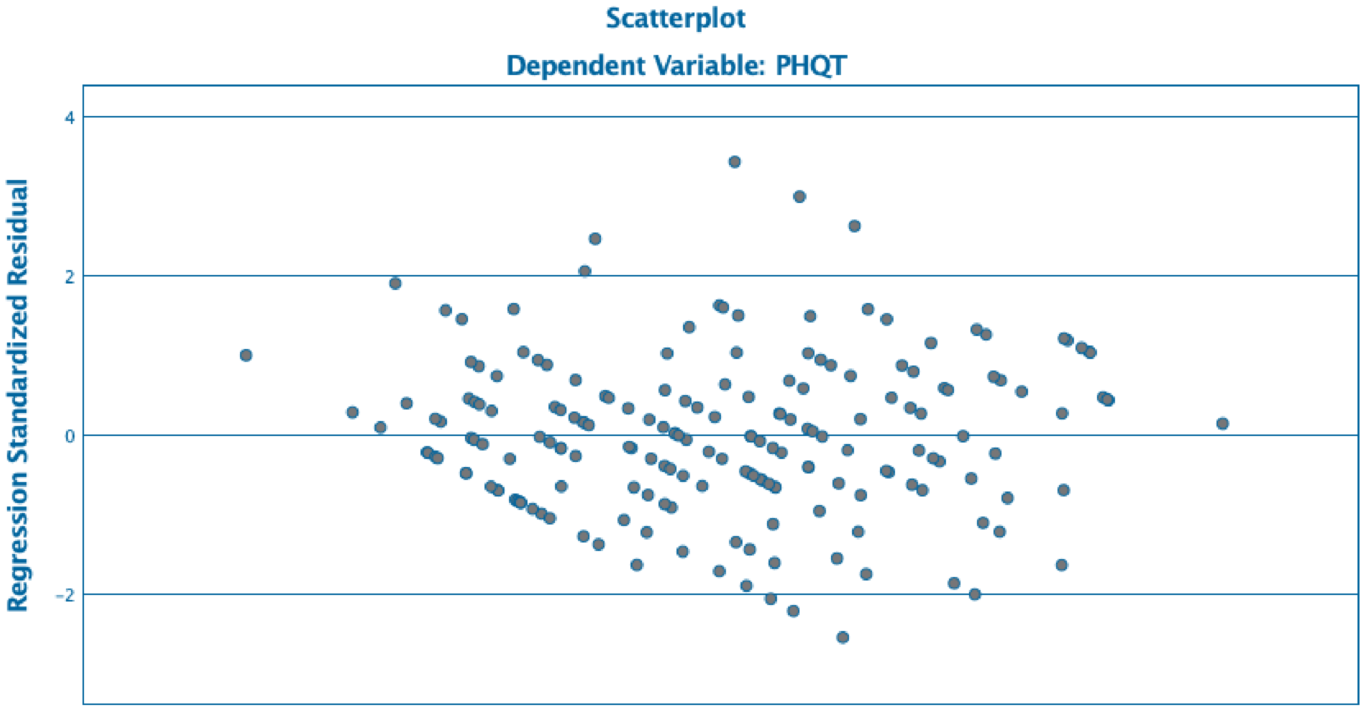

Supplement: Supplementary file 1 [file Data_Sheet_1.pdf]
